# Using Multisource Data to Assess PM_2.5_ Exposure and Spatial Analysis of Lung Cancer in Guangzhou, China

**DOI:** 10.3390/ijerph19052629

**Published:** 2022-02-24

**Authors:** Wenfeng Fan, Linyu Xu, Hanzhong Zheng

**Affiliations:** School of Environment, Beijing Normal University, No. 19, Xinjiekouwai Street, Haidian District, Beijing 100875, China; 201921180045@mail.bnu.edu.cn (W.F.); zhenghz_2016@163.com (H.Z.)

**Keywords:** ordinary kriging interpolation model, spatial correlation analysis, PM_2.5_ pollution exposure, health risk

## Abstract

Elevated air pollution, along with rapid urbanization, have imposed higher health risks and a higher disease burden on urban residents. To accurately assess the increasing exposure risk and the spatial association between PM_2.5_ and lung cancer incidence, this study integrated PM_2.5_ data from the National Air Quality Monitoring Platform and location-based service (LBS) data to introduce an improved PM_2.5_ exposure model for high-precision spatial assessment of Guangzhou, China. In this context, the spatial autocorrelation method was used to evaluate the spatial correlation between lung cancer incidence and PM_2.5_. The results showed that people in densely populated areas suffered from higher exposure risk, and the spatial distribution of population exposure risk was highly consistent with the dynamic distribution of the population. In addition, areas with PM_2.5_ roughly overlapped with areas with high lung cancer incidence, and the lung cancer incidence in different locations was not randomly distributed, confirming that lung cancer incidence was significantly associated with PM_2.5_ exposure. Therefore, dynamic population distribution has a great impact on the accurate assessment of environmental exposure and health burden, and it is necessary to use LBS data to improve the exposure assessment model. More mitigation controls are needed in highly populated and highly polluted areas.

## 1. Introduction

Air pollution is a key factor threatening human health. The Global Burden of Disease Study in 2020 shows that in 2019, the death toll of females and males attributable to air pollution ranked third among all factors. It reached 2.92 million and 3.75 million, which accounted for 11.3% and 12.2% of the total deaths, respectively [1]. China’s death rate due to particulate pollution in 2019 was 125.61 per 100,000 people. Long-term exposure to high concentrations of PM_2.5_ will increase the morbidity and mortality attributable to respiratory and cardiovascular diseases and lung cancer. Although smoking is the largest contributor to lung cancer, environmental pollutant exposures has also been identified as a key risk factor for lung cancer [2,3]. Compared with other air pollutants such as sulfur dioxide and nitrogen oxides, PM_2.5_ has been regarded as an important carcinogen. The WHO has classified smog as a first-degree carcinogen, which is equivalent to determining that there is sufficient evidence at present showing that air pollution and cancer (lung cancer) have a direct causal relationship. The relative risks of lung cancer incidence and mortality following exposure to PM_2.5_ were 1.08 (95% CI: 1.03, 1.12) and 1.11 (95% CI: 1.05, 1.18), respectively [4,5,6,7]. A follow-up survey of 313,000 people in nine European countries shows that chronic low-level exposure (limited to annual concentration < 10 μg/m^3^ or daily concentration <30 μg/m^3^ and (365 day moving average) <10 μg/m^3^) increases the risk of lung cancer [8,9]. The nervous system and cognitive ability are also negatively affected [10,11,12,13]. These findings are particularly worrying in China. Currently, urban air pollution in China has become a serious problem. As a representative megacity, Guangzhou’s cancer incidence in 2015 (299.10/100,000) was much higher than the world average (198.40/100,000), and the number one lung cancer incidence in the city was lung cancer. Closely connected with air pollutants. In 2015, the annual average concentration of PM_2.5_ exceeded 40 μg/m^3^ [14], approximately four times higher than the WHO recommended air quality standard. Therefore, an accurate assessment of the current PM_2.5_ exposure of Guangzhou residents is the key to formulating corresponding countermeasures.

Accurate estimation of PM_2.5_ concentrations is a prerequisite for PM_2.5_ exposure assessment and epidemiological correlation analysis. Routine monitoring of PM_2.5_ started in China in 2013. This helps residents preliminarily understand the real-time air pollution quality level. However, it cannot effectively estimate the huge spatial variability of pollutant concentrations. Air pollution modeling can be an effective approach with two commonly used methods for capturing ambient air pollution gradients being spatial interpolation and land-use regression (LUR), which are widely used to predict the relationship between PM_2.5_ exposure and health effects. Spatial interpolation uses monitoring data from the limited number of stations to predict large-scale spatial pollutant concentration changes. Compared with spatial interpolation, the LUR model can integrate potential geographic predictive variables into the development of multiple linear regression models in geographic information systems (GIS), but land-use regression has limited ability to capture temporal changes. The commonly used variables include land-use type, population size, traffic conditions, meteorological conditions, and pollution source locations [15,16,17,18]. POI (point of interest) data from recent studies can also be used as predictors [19,20].

In addition, there is a mismatch between the spatial distribution of PM_2.5_ concentration and the population density. Prediction of reliable exposure levels is needed to take the population distribution into consideration. The current demographic data based on the census provide reliable population distribution information that has been verified by multiple studies [20,21,22]. These data are commonly used to calculate the population-weighted exposure level of the age, education level, and income level subgroups [23,24,25,26], which makes it possible to assess and visualize the risk of residential exposure. To more accurately understand the exposure situation during population movement, researchers also collect LBS data of population movement information to verify the corresponding results.

Currently, the study of the impact of the PM_2.5_ concentration on disease and health focuses on the calculation of the population-weighted concentration for subgroups of different ages and education levels. The population distribution of different subgroups, the dose-response relationship of human health, and the PM_2.5_ concentrations were combined and then toxicologically analyzed to obtain the actual exposure risk. Another focus limits the analysis to cohort studies. By analyzing the temporal statistical relationship between historical PM_2.5_ monitoring data and the incidence of cardiopulmonary disease, the relationship between the two can be derived. However, from the urban perspective, the overall extent of the spatial correlation between PM_2.5_ and lung cancer incidence is limited. Therefore, the objectives of this study are to (1) select a proper model for studying urban-scale spatial pollution levels with limited pollutant monitoring data to achieve spatial visualization and use portable monitoring equipment to verify the reliability of the model used in monitoring data; (2) study the risks of static and dynamic population exposure to PM_2.5_ and obtain the difference between urban centers and surrounding areas. (3) Based on the available lung cancer incidence data and the ground PM_2.5_ air concentration for spatial statistical analysis, this study’s aim was to explore the spatial correlation between PM_2.5_ and the high incidence of lung cancer and to assess the health risks of residents in different areas of the city exposed to pollutants. This would advance China’s environmental and epidemiological research and contribute to the development of effective measures to alleviate the current exposure risks.

## 2. Materials and Methods

### 2.1. Study Area

**Figure 1:**
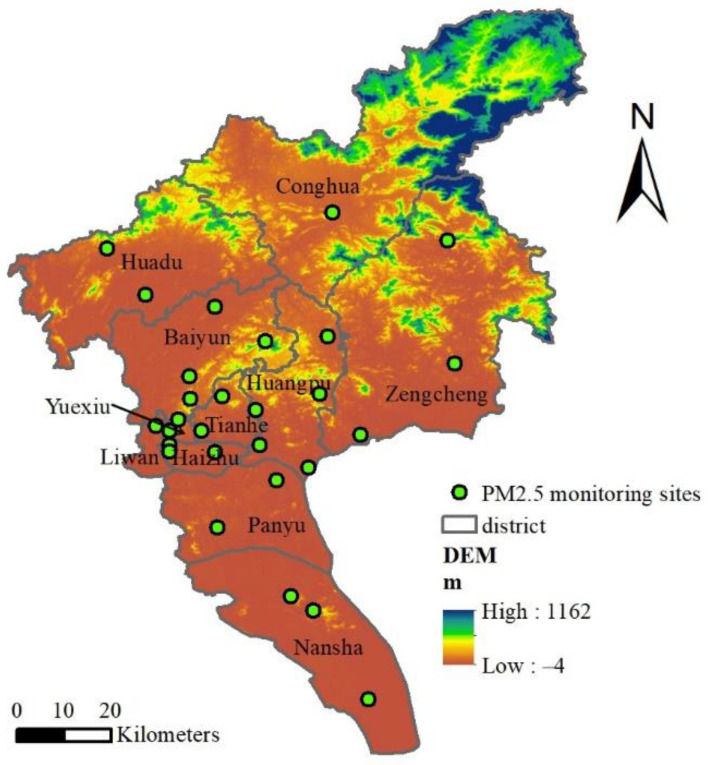
Spatial distribution mapping of air monitoring stations in Guangzhou.

The Pearl River Delta, where Guangzhou is located, is one of the most urbanized and industrialized regions in the world. As the core city of the Pearl River Delta, Guangzhou, a megacity with a population of more than 12.7 million [27], consists of 11 district-level administrative regions and 171 townships and blocks (In the schematic diagram of this research, DISTRICT refers to the district-level administrative district of Guangzhou, and BLOCK BORDER refers to the block-level administrative district of Guangzhou), which covers an area of approximately 7434.4 km^2^. It is one of China’s main economic and manufacturing core areas; its main industries are chemical products, biotechnology, electronic and communication equipment manufacturing, as well as food, motor, automobile, and metal manufacturing [28]. The rapid development of industrialization activities has led to a sharp deterioration in the air quality in Guangzhou. Vehicle emissions, secondary aerosols, biomass combustion emissions, sea salt and coal combustion are all important factors that affect Guangzhou’s atmospheric PM_2.5_ [29]. Therefore, it is necessary to carry out PM_2.5_ spatial exposure research in Guangzhou. Figure 1 shows the location of Guangzhou city and its 28 major air quality monitoring stations in 2015.

### 2.2. Data Source for Model Development

As the dependent variable for the study, the dataset of the daily hourly PM_2.5_ concentration measurement values of the 28 monitoring stations in Guangzhou in 2015 and 2021 were collected from the National Urban Air Quality Real-Time Publishing Platform and the Guangzhou Ecological Environment Bureau. Maofengshan Forest Park Station served as a background station. The daily measurement values were summarized and converted into the annual average and seasonal average of the development model. To verify the spatial distribution results of the model, the researchers went to Guangzhou Yuexiu District, Tianhe District, Panyu District, Huangpu District, Conghua District, and Zengcheng District and selected typical blocks to monitor the concentration of fine particles on the spot from 23–29 September 2021. The concentration data were 9000 pieces, and the weather data were approximately 18,000 pieces. At the same time, we collected Weibo sign-in data in September 2015 and 2021 through Python on the Weibo API (http://open.weibo.com). A total of approximately 270,000 pieces of information were obtained from the LBS data, including user ID (anonymous), release time and location, and these data were used to draw a population dynamics distribution map to compare and analyze the difference and connection between the population dynamics distribution on working days and non-working days.

### 2.3. Spatial Analysis Methods

LUR and ordinary kriging are often used to visualize the spatial distribution of PM_2.5_ concentrations. Both methods are based on ArcGIS (Manufactured by Environmental Systems Research Institute, Redlands, CA, USA) to predict and analyze high-resolution aerosol pollutant levels. Due to the limited monitoring data in the study area, this study used both methods to estimate the spatial distribution of the PM_2.5_ concentrations in the entire urban area and compared their accuracy.

LUR is a multivariate simulation method optimized by the least square method to predict the pollution concentration at any point in the space. The model can integrate various factors that have potential effects on the PM_2.5_ concentration. This study used the annual average PM_2.5_ concentration of 28 air quality monitoring stations in Guangzhou in 2015 as the dependent variable. The independent variables were the area of land use, the length of the road network, and the population density in the study area, as well as the annual average temperature, wind speed, elevation, and population density. Multiple regression analysis was performed. All the data were preprocessed by ArcGIS into vector files that could be spatially analyzed. The Supplementary Material explains the specific process of the LUR derivation.

Kriging estimates the level of pollutants based on environmental monitoring information. In this study, ordinary kriging was used to spatially interpolate the PM_2.5_ data collected from 28 major sites in 11 districts of Guangzhou, and a spherical spatial model with the weighted least squares method was used to generate the spatial distribution of the PM_2.5_ concentration (1 km × 1 km). 

Based on R^2^, the relative error (RE), and root mean square error (RMSE), the model performances of the two methods were compared. At the same time, cross-validation was performed for external verification. Model training data were used from 27 monitoring stations, and the remaining dataset was used as a testing dataset, with 28 iterations performed. The error between the measured value and the estimated value was estimated to confirm the model robustness.

### 2.4. PM_2.5_ Exposure and Health Risk Assessment

Previous studies have shown that there is a regional deviation between the population distribution of the city and the distribution of the PM_2.5_ concentration [30]. The same concentration of PM_2.5_ pollution can result in far more serious health hazards in densely populated areas than sparsely populated areas.

To compare the results of the static and dynamic population models and understand the exposure during population movement, the researchers collected census data and dynamic population information LBS data, aggregated all the geotag records of each grid, redistributed the population data of Guangzhou, and used the population under multiple time scales as weights. The corresponding PM_2.5_ trend surface and the number of people in the grid were superimposed in ArcMap, the PM_2.5_ concentration in the center of the grid and the number of people in the grid were extracted. Finally, the population exposure risk assessment model [29] was used to calculate the Guangzhou area at the 2015 PM_2.5_ pollution exposure level, draw the PM_2.5_ exposure risk distribution map to estimate the static and dynamic exposure of the population to PM_2.5_, make the time resolution consistent with the population map based on the LBS [30], and measure the actual exposure level of the population. The model is as follows:

$$E_{i}=P_{i}\times C_{i}$$

where, *E_i_* is the population weighted exposure level of the *i*-th pixel, *C_i_* is the PM_2.5_ concentration of the *i*-th pixel, *P_i_* is the estimated population in the *i*-th pixel, and *n* is the total number of pixels.

### 2.5. The Spatial Relationship between PM_2.5_ and the Incidence of Cardiopulmonary Diseases

Spatial autocorrelation analysis was used to evaluate the spatial relationship between the incidence of lung cancer (the number of new malignant tumor cases per 100,000 people registered in a place in a certain year) and PM_2.5_ in Guangzhou from 2013 to 2015 and to explore the synchronous and lagged association between the two by the correlation analysis method. This study collected data on the incidence of lung cancer (1/100,000) in 11 districts of Guangzhou from 2012 to 2015. These data are mainly derived from the “Guangzhou Cancer Registration Annual Report” compiled by 120 online hospitals with tumor diagnosis and treatment capabilities, and the follow-up summary of 210 community health service agencies in the city and use Percentage of microscopic verification, Percentage of death certificates and Mortality to incidence ratio quality indicators. Spatial interpolation was used to obtain the incidence data for an average of 15 blocks in each district. ArcGIS was used to assign these data to the centroid of the district and then perform ordinary kriging interpolation [31], with the lung cancer incidence distribution data of the entire city (resolution: 1 km × 1 km) being obtained thereafter. Moran’s I index and standardized statistic Z were then calculated for the incidence of lung cancer in the study area, both of which were used to estimate the spatial clustering of the entire area and test the spatial autocorrelation between PM_2.5_ and the incidence of lung cancer. Global Moran’s I is used as a spatial autocorrelation measurement, calculated as follows. If the data are nonrandomly distributed, it means that the spatial location has an impact on the incidence of lung cancer.

$$I=\frac{\sum_{i=1}^{n}\sum_{j}^{n}w_{ij}\left(x_{i}-\bar{x}\right)\left(x_{j}-\bar{x}\right)}{s^{2}\sum_{i=1}^{n}\sum_{j=1}^{n}w_{ij}}$$

where *x_i_* and *x_j_* is the lung cancer incidence or mortality of each area polygon, *s*^2^ is the sample variance, *n* is the number of area polygons, and *w_ij_* is the spatial weights matrix, which defines the local neighborhood around each area polygon, which is created by autocorrelation measurement.

Previous studies have found that the impact of PM_2.5_ concentration levels on the incidence of lung cancer in the population was the result of long-term effects [3,32], and there may be a lagging effect. However, there is no definite duration of PM_2.5_ exposure on the incidence of lung cancer. The incidence of lung cancer in different regions of China has a delay in response to PM_2.5_ exposure ranging from 5 to 8 years [33,34]. We obtained the annual average concentration of PM_2.5_ near the surface of China’s districts and counties before 2013 from the dataset website established by the atmospheric composition analysis group of Dalhousie University in Canada (https://sites.wustl.edu/acag/datasets/surface-pm2-5/, accessed on 31 December 2021). The correlation between the PM_2.5_ concentration levels in the 2005–2015 time series and the incidence of lung cancer (2012–2015) was investigated. It was hoped that this would derive the duration of PM_2.5_ exposure in Guangzhou that can affect the incidence of lung cancer.

## 3. Results

### 3.1. The Spatial Distribution Characteristics of PM_2.5_

**Figure 2:**
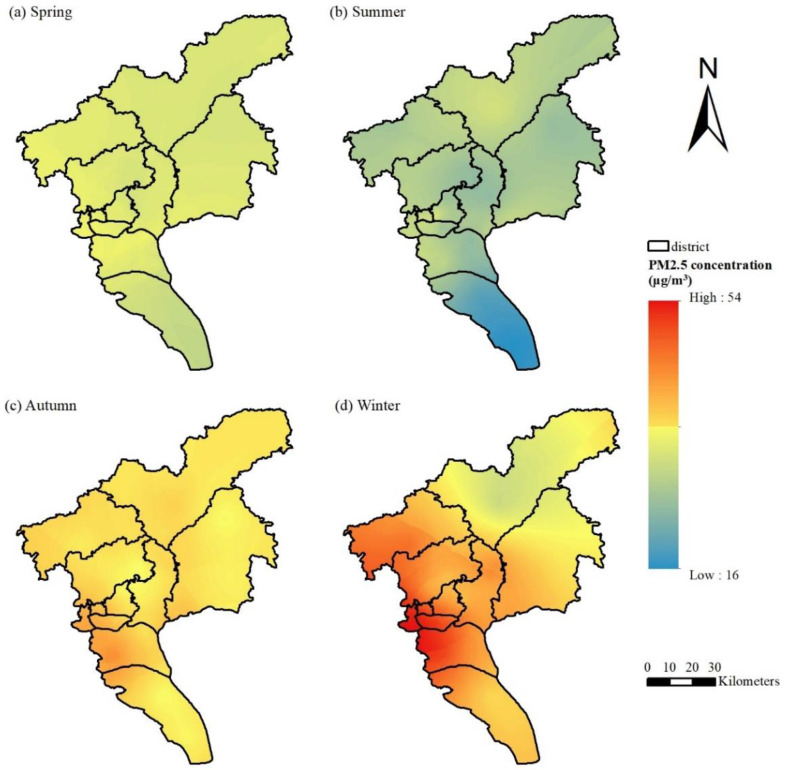
Spatial distribution of PM_2.5_ through ordinary kriging in 2015: (**a**) spring, (**b**) summer, (**c**) autumn, (**d**) winter.

**Figure 3:**
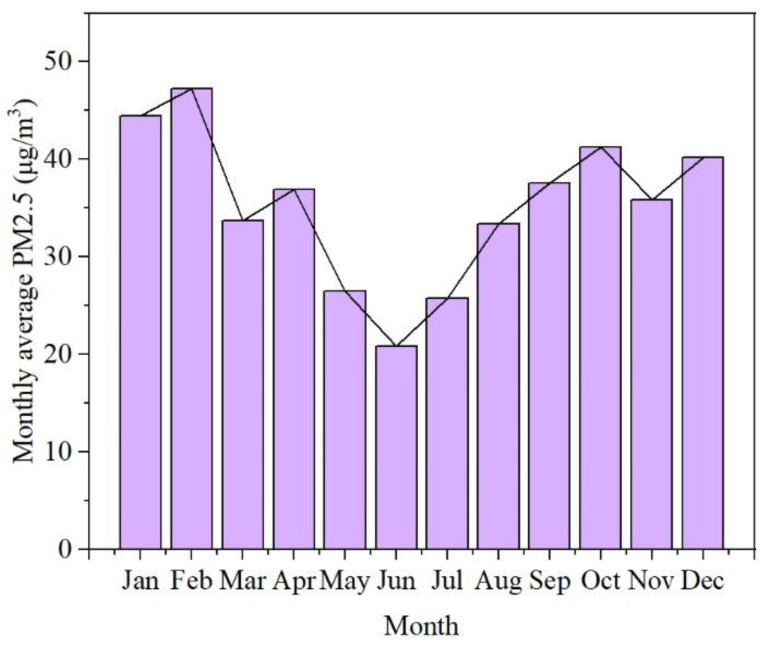
Monthly average concentration of PM_2.5_ in Guangzhou from January to December 2015.

The comparison of the two spatial prediction models has been explained in the Supplementary Material. The prediction value of the ordinary kriging spatial interpolation is similar to that obtained by the LUR model, but it performs better in predicting the annual and seasonal averages. Therefore, in this section, ordinary kriging interpolation was used for the daily hourly PM_2.5_ concentration data in Guangzhou to obtain the spatial distribution. The average PM_2.5_ concentration in Guangzhou in 2015 was 35.34 μg/m^3^. As shown in Figure 2, the distribution of PM_2.5_ in Guangzhou presents obvious temporal and spatial heterogeneity. The PM_2.5_ concentration in the study area was high in the west and low in the north and south, which is consistent with the topography of Guangzhou, which is high in the northeast and gentle in the middle. The Liwan District, Yuexiu District, Haizhu District, and the northern part of the Panyu District in the urban cores are the centers of PM_2.5_ pollution in Guangzhou, with an average annual concentration of approximately 40 μg/m^3^. They were followed by Huadu District and the western part of Baiyun District. The level of PM_2.5_ pollution in the Conghua District in the north and the Nansha District in the south was relatively low, with an average annual rate of only 32 μg/m^3^, which is lower than the Chinese environmental quality standard of 35 μg/m^3^ (Level II); this indicates good air quality. As shown in Figure 3, the PM_2.5_ concentration from January to December 2015 showed a trend of first declining and then increasing thereafter. The PM_2.5_ concentration was highest (>44 μg/m^3^) in January and February, while was lowest (20 μg/m^3^) was in June. Obviously, there is a significant variation in the PM_2.5_ concentration between months, and the concentration of PM_2.5_ in winter (44.10 μg/m^3^) followed by autumn (40.04 μg/m^3^) is significantly higher than in other seasons, while the PM_2.5_ concentration levels in spring (32.38 μg/m^3^) and summer (26.65 μg/m^3^) were lower, as these are the seasons that have better air quality. The spatial distribution of the seasonal PM_2.5_ was obtained after spatial interpolation (Figure 2). The northwestern part of Guangzhou has high PM_2.5_ levels in spring and summer, while the pollution centers in autumn and winter are concentrated in the urban and industrial areas. In summer, the PM_2.5_ concentration is generally not high, and the areas with high concentrations are increasingly prominent.

As shown in Table 1, the on-site monitoring results of PM_2.5_ concentrations in six districts of Guangzhou from 23 to 29 September 2021 showed that the PM_2.5_ concentration of Tianhe (41.85 μg/m^3^) and Yuexiu (41.13 μg/m^3^) in the core area of the city were the top two, and the highest value is more than 150 μg/m^3^, followed by Zengcheng (41.91 μg/m^3^), Panyu (41.08 μg/m^3^) and Huangpu (37.62 μg/m^3^) are slightly lower than this, and Conghua (33.46 μg/m^3^) is the lowest, which is lower than the national secondary standard limit, and the spatial distribution is similar to model prediction, which directly proves the reliability of the prediction results.

### 3.2. Population Distribution and PM_2.5_ Exposure Level

**Figure 4:**
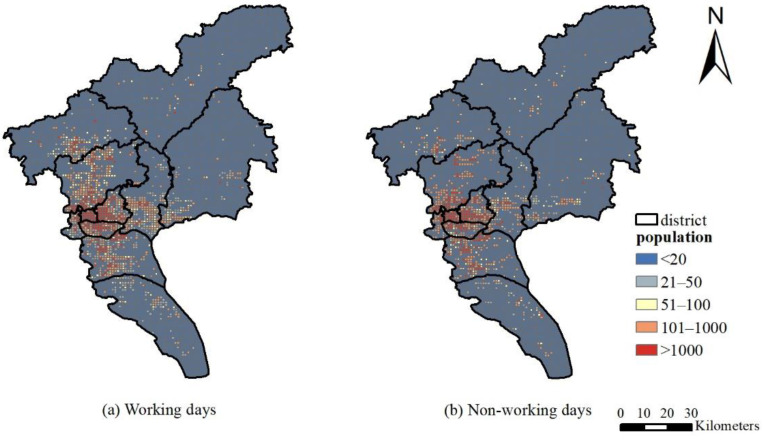
Dynamic population distribution in September 2021: (**a**) working days; (**b**) non-working days.

The administrative map of the towns and blocks in Guangzhou was divided into a grid of 1 km × 1 km. The population data of 171 blocks in 11 districts of Guangzhou were obtained from the sixth national census in 2010, and these data were added to the grid to draw the population distribution map of Guangzhou in 2015 consistent with the PM_2.5_ spatial interpolation resolution. As shown in Figure 4, Guangzhou’s densely populated areas are concentrated in the western core of the Liwan District, Haizhu District, and Yuexiu District, followed by the Tianhe District, Panyu District, and Huangpu District. The sparsely populated areas are in the south and north. At the same time, we integrate LBS data and district/county-scale population data to draw the population distribution of working days and non-working days in 2021 in Guangzhou (Figure 4). The stretched colors from dark blue to red indicate different population distributions. The results show that in Guangzhou, the vast majority of the population is concentrated in the urban core area, and the rest are distributed throughout the entire area. For areas where hotspots are concentrated, LBS data can provide a clear visualization of population distribution, showing that non-urban core areas also have hotspot-concentrated areas.

**Figure 5:**
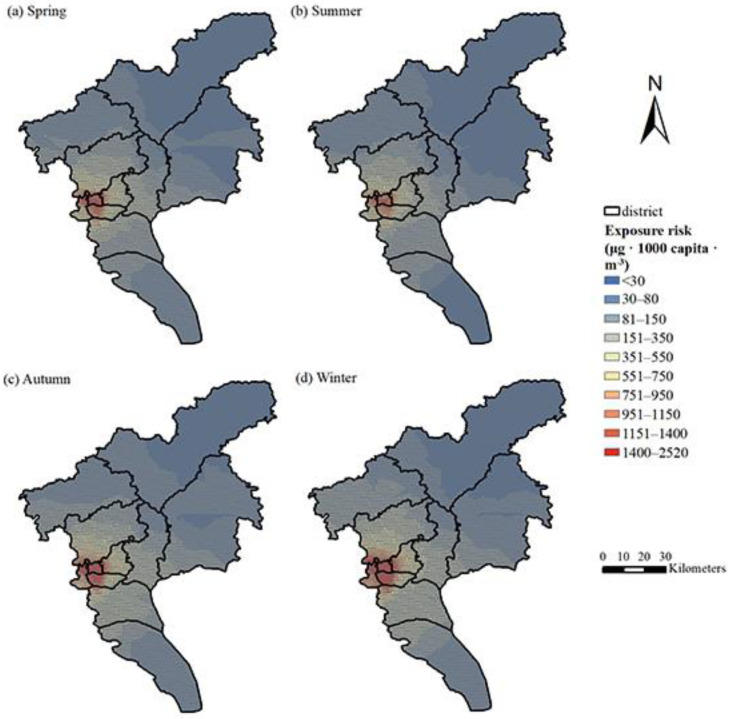
Exposure risk of PM_2.5_ in Guangzhou across the four seasons of 2015: (**a**) spring; (**b**) summer; (**c**) autumn; (**d**) winter.

The distribution of Guangzhou’s population exposure intensity in 2015 is shown in Figure 5. The areas with high average exposure risk in 2015 were concentrated in the Liwan District, Yuexiu District, Haizhu District, and Tianhe District of Guangzhou City. In contrast, the Conghua District and Zengcheng District have lower exposure risks.

With regard to the seasonal exposure risk, the population exposure risk is highest in winter, followed by autumn. The northern part of the Nansha District Guangzhou City, the Baiyun District, Panyu District, Huangpu District, and downtown Guangzhou are all areas with higher population exposure risks in winter. In the northern part of the Nansha District and Huadu District, where the PM_2.5_ levels are relatively high in winter, these areas have a relatively small population, and the exposure risk is lower than that of the urban-core area. The risk of pollution exposure in autumn is between that of winter and spring. The exposure risk in spring and summer is low, which are the seasons with the lowest health risk of the year. The higher-risk areas are also concentrated in the urban core. In the southern part of the Nansha District, Zengcheng District, and Conghua District, the exposure risks in the four seasons were relatively lower.

**Figure 6:**
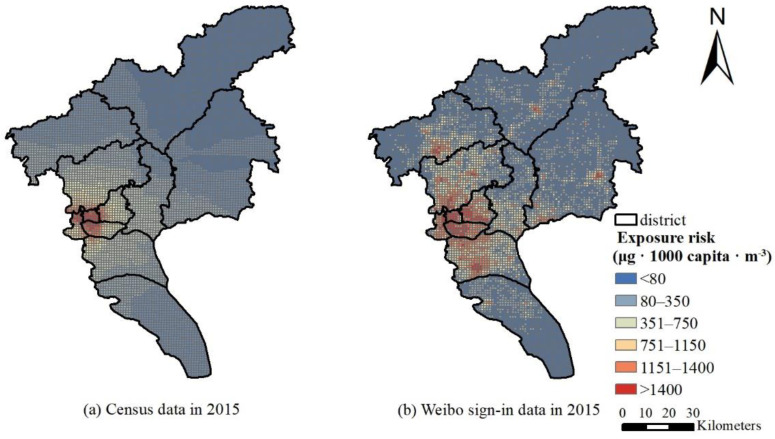
Comparison of average annual PM_2.5_ population exposure risk in Guangzhou: (**a**) census data in 2015; (**b**) Weibo sign-in data in 2015.

**Figure 7:**
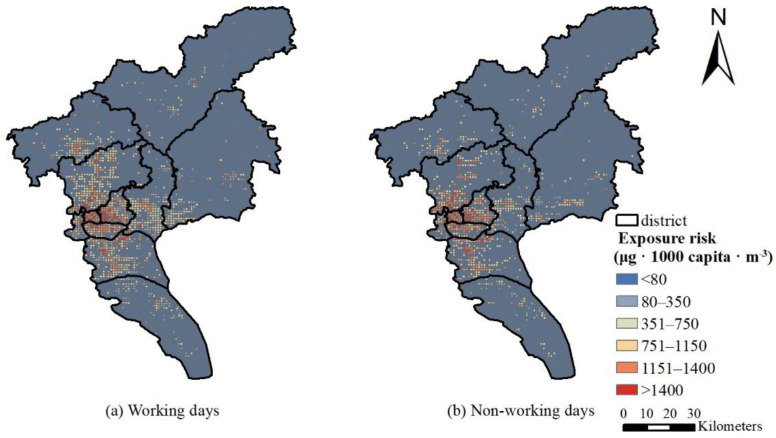
Population exposure risk distribution in September 2021: (**a**) working days; (**b**) non-working days.

In comparison, the 2015 annual population exposure risk based on LBS data is quite different from the static population exposure in terms of spatial characteristics. The static population exposure distribution in Figure 6a cannot accurately depict spatial heterogeneity, while the hot spots of dynamic population exposure are scattered. In addition to the four districts in downtown Guangzhou, Baiyun airport, the chime long and university town of Panyu, business circle and government surrounding of Conghua and Zengcheng are all hot areas, but the static population exposure does not reflect these hot spots. Comparing the exposure risks on working days and non-working days, as shown in Figure 7, people on working days mainly gather in major office areas and business districts, mainly in the four districts of the city. Although the four districts of the city are still hotspots on non-working days, the Guangzhou Tower, Tianhe Sports Commercial District, and Huangpu Science City, where the crowds are relatively concentrated on the working day, are scattered into multiple hotspots. Due to the diverse locations to travel for non-working days, the distribution in each district is more scattered.

The spatial interpolation comparison with the annual average concentration of PM_2.5_ reveals that the areas with high PM_2.5_ concentrations are not exactly the same as the areas with high exposure risks. Compared with the densely populated urban areas, the Panyu District and the western part of the Baiyun District, which are also areas with high PM_2.5_ concentrations, have higher exposure risks than those in the city cores, which verifies the necessity of considering the intensity of the population exposure. When we examine the population density distribution map, it shows that the exposure risk distribution is very similar to the population distribution, which indicates that the level of the exposure risk is closely related to the population density.

### 3.3. The Spatial Correlation between the PM_2.5_ Levels in Guangzhou and the Incidence of Lung Cancer

**Figure 8:**
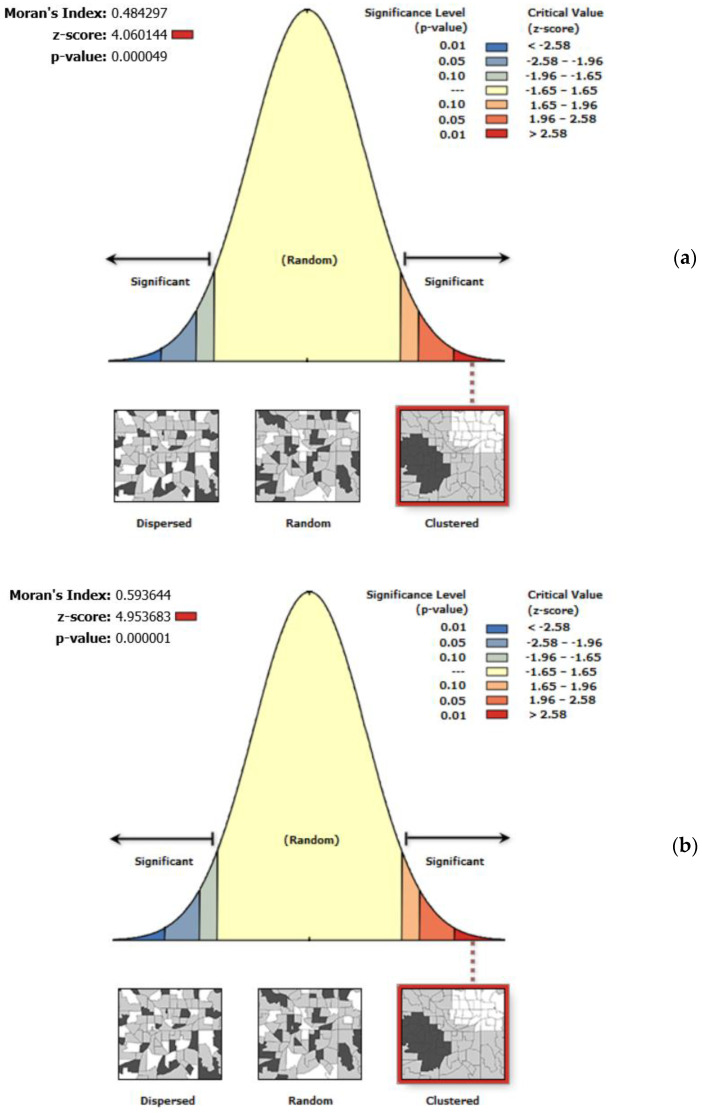
Moran’s I index and Z values of lung cancer incidence in Guangzhou: (**a**) 2015; (**b**) 2013.

**Figure 9:**
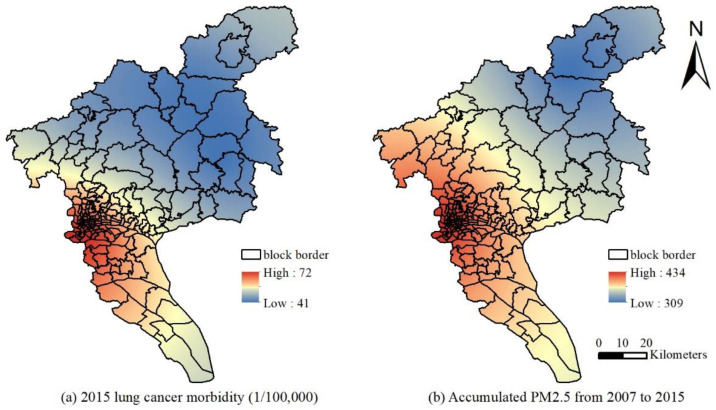
Spatial distribution of lung cancer incidence in 2015 and the total concentration of PM_2.5_ in 2007–2015: (**a**) 2015 lung cancer morbidity (1/100,000); (**b**) accumulated PM_2.5_ from 2007 to 2015.

**Figure 10:**
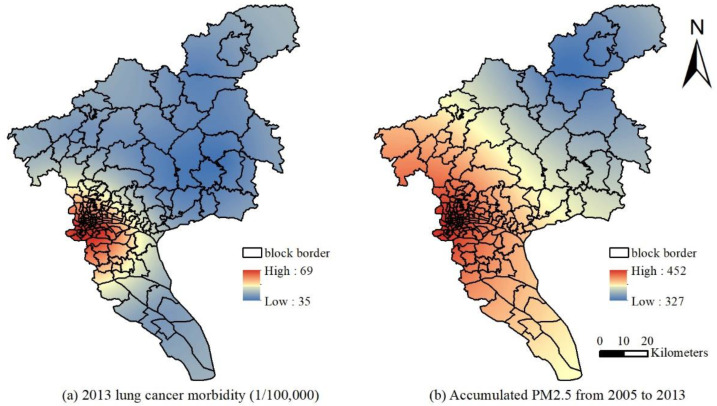
Spatial distribution of lung cancer incidence in 2015 and the total concentration of PM_2.5_ in 2005–2013: (**a**) 2013 lung cancer morbidity (1/100,000); (**b**) accumulated PM_2.5_ from 2005 to 2013.

This study uses the global Moran’s I index to verify and judge the spatial distribution status and clustering model of the incidence of lung cancer in the block areas of Guangzhou from 2014 to 2015 and 2012 to 2013. The statistic, |Z| > 1.96 (or >4), indicates that the spatial autocorrelation of the data in a region is significant at a confidence level of *p* = 0.05 (or *p* = 0.01). The data exhibit regularity in space and do not follow a random distribution [31,35]. Figure 8 shows the results of the spatial autocorrelation. The Moran’s I index and standardized statistics in 2013 and 2015 were 0.5936 and 4.9537 and 0.4843 and 4.0601, respectively. Therefore, the incidence of lung cancer has a significant spatial autocorrelation with environmental factors, and the incidence of lung cancer in different locations throughout the region was not randomly distributed. Furthermore, the correlation between the average PM_2.5_ concentration of the current year and the previous eight years and the incidence of lung cancer were determined using SPSS 13.0. After the normality test by the K-S method, Pearson correlation analysis was performed. The correlation coefficients were 0.743, 0.757, 0.768, 0.784, 0.717, 0.775, 0.806, 0.741, and 0.774 from 2005 to 2013, respectively. The correlation coefficients in 2015 and the previous 8 years were 0.767, 0.781, 0.748, 0.731, 0.816, 0.717, 0.792, 0.786, and 0.816, respectively. The results suggest that the incidence of lung cancer was significantly correlated with the PM_2.5_ concentration level at the *p* = 0.01 level in the current year and the previous eight years. Kriging interpolation with the data of 11 district-level administrative regions from 2014 to 2015 and 2012 to 2013 was used to obtain the spatial distribution of the lung cancer incidence and PM_2.5_ cumulative concentration (Figure 9 and Figure 10). The locations of high PM_2.5_ levels and the high incidence of lung cancer were roughly the same. The overall high PM_2.5_ concentration was in the core of the city, and it was low in the areas far from the urban core, high in the southern and central parts of the city, and low in the northern part of the city.

## 4. Discussion

### 4.1. Factors Affecting the Spatial Distribution of PM_2.5_

The concentration of PM_2.5_ in Guangzhou is significantly lower than that of cities in northern China (the main reason is the difference in weather conditions) [14,36]. The overall distribution characteristics are high in the west and low in the north and south. The Liwan District, Yuexiu District, Haizhu District, and the northern part of the Panyu District in the core area of the city are PM_2.5_ pollution centers. This kind of PM_2.5_ pollution level and distribution are mainly due to various factors, such as traffic, meteorological conditions, and the distribution of pollution sources [37]. On the one hand, seasonal climatic conditions may have contributions. The temperature in autumn is low, and a stable atmosphere is not conducive to the dilution and diffusion of pollutants. Moreover, the frequency and intensity of temperature inversions are high, and the duration is long. The winter climate is dry, with more wind and less rain, which is favorable for dust conditions. Therefore, the average concentration of PM_2.5_ in autumn and winter is high. In addition, spring is the East Asian rainy season in Guangzhou. The lowest average PM_2.5_ concentration occurs in summer as a result of the deposition of rain on pollutants, higher temperatures, and low atmospheric stability together with the concentrated rainfall events that are conducive to the diffusion, wet deposition, and dilution of atmospheric pollutants [38]. On the other hand, Guangzhou’s industrial zones are concentrated in the Huadu, Baiyun, and Nansha Districts, and major urban polluting companies are concentrated in these areas. The southwestern part of Guangzhou is close to the traditional heavy industrial cities of Foshan and Zhongshan. These cities have developed industries, such as building materials, ceramics, and hardware, with large pollutant emissions. It is easy for the PM_2.5_ concentration of the surrounding cities to be affected under unfavorable meteorological conditions and cause the air quality to deteriorate [39,40].

On-site monitoring of PM_2.5_ concentrations showed that the PM_2.5_ concentration distribution trend of Guangzhou’s six districts was generally consistent with the model results. The maximum value of actual measurement data in Zengcheng District exceeded 300 μg/m^3^, indicating that large-scale straw burning in the suburbs of Guangzhou in late summer and early autumn caused particulate pollution to escalate; after verification of meteorological conditions, the monitoring period was one week after the typhoon on 23–29 September, and the PM_2.5_ concentration showed a trend from low to high. The daily average concentration of Tianhe and Yuexiu in the core areas even exceeded 80 μg/m^3^. These daily changes may be caused by changes in meteorological factors (such as wind direction, temperature, mixing height, etc.). These factors change with the day and significantly affect the formation, diffusion and removal mechanism of air pollution [41]. In addition, the temporal heterogeneity of the PM_2.5_ concentration may be affected by road location and layout, and traffic dynamics (flow, speed, fleet composition) [42]. These typical sampling units provide high-quality air pollutant data, which show more geographic differences than the data captured by conventional monitoring.

### 4.2. Population Exposure Assessment and Its Lung Cancer Health Risk

The results of the study show that using exposure level indicators that consider population distribution is more robust than simply using PM_2.5_ concentration to assess population pollutant exposure levels.

The pixel-based dynamic population map can appropriately depict the population exposure risk distribution. Compared with previous exposure assessment methods, the proposed method fully considers the estimation accuracy of PM_2.5_ concentration and the temporal and spatial variability of population distribution. In addition, the average level of PM_2.5_ exposure in each grid is determined by the population distribution of the time scale because the population distribution of different time scales has a greater degree of variation. Since the distribution of the static population at various time scales remains unchanged, the use of census data to calculate the temporal and spatial differences of PM_2.5_ exposure levels depends on the PM_2.5_ concentration, and the use of LBS data depends more on the population distribution during the period. The pollutant exposure level of each grid was tested for normality, and all did not conform to the normal distribution. The Mann-Whitney U test was used to analyze the difference between dynamic and static exposure levels. The results showed that there was a significant difference between the static population and dynamic population exposure levels (*p* < 0.001). Therefore, the use of dynamic population distribution data to analyze population exposure levels is more accurate, and the estimated health risks are also more accurate.

According to the results of the population exposure risk assessment model, Yuexiu, Tianhe, Liwan and other areas have high long-term exposure risks. These areas have a large population of people for a long time, and they are near important transportation links and hubs. Human activities such as automobile emissions have led to higher overall pollutant concentrations. In addition, the areas with higher PM_2.5_ exposure levels in any scenario are the Haizhu District and the northwestern part of Panyu, which are related to the high levels of both the number of people and the concentration.

On the other hand, there is strong evidence that acute and long-term exposure to PM_2.5_ increases the incidence of heart and lung diseases. For every 10 μg/m^3^ increase in PM_2.5_ concentration, lung cancer mortality will increase by 15–27% [3]. In China, high levels of indoor air pollution caused by the burning of coal and biomass have also led to a high incidence of lung cancer even among non-smokers [43]. The incidence of lung cancer is affected by many factors, with smoking, special occupational exposure, air pollution, and genetic factors being the main factors. According to the Guangzhou Tobacco Survey, in 2004, the smoking rate of residents over 15 years old in Guangzhou was 28.4%, in 2010 it was 26.7%, and in 2017 it dropped to 20.7%. Obviously, the number of smokers has been declining in the past ten years. With the continuous improvement of public health awareness, the smoking rate will be further reduced. The main cause of lung cancer will be air pollution, especially fine particulate matter.

Spatial interpolation was performed for lung cancer incidence data in 11 administrative districts of Guangzhou based on ArcGIS for spatial autocorrelation analysis. Taking into account the lag effect between the PM_2.5_ levels and lung cancer, the correlation analysis was performed, and the results suggest that the incidence of lung cancer in 2012–2015 was significantly related to the PM_2.5_ concentration levels of the current year and the previous 7–8 years. The results also show that the PM_2.5_ levels in this period of 4–6 years have a significant impact on the incidence of lung cancer. The spatial interpolation results also show that the high incidence of lung cancer is concentrated in the urban area of Guangzhou. It shows a decreasing trend from west to east, and it is lower in the southern and northern parts, which is generally consistent with the spatial distribution pattern of the cumulative concentration of PM_2.5_. Considering the cumulative and lagging effects of the PM_2.5_ pollution levels on the incidence of lung cancer from the time and space scales, the study focused on the impact of historical factors on the incidence of lung cancer. The results indicate that the incidence of lung cancer does have a certain correlation with PM_2.5_, which is consistent with Han [44].

### 4.3. Research Advantages and Limitations

This study uses limited monitoring data and spatial pollution models to draw a spatial distribution map of Guangzhou’s annual and quarterly average PM_2.5_ in 2015. On-site monitoring is combined to verify the results with innovation. The results show that the model simulation is reliable. In assessing population exposure levels, the innovative use of LBS data to draw dynamic population distribution, fully considering the temporal and spatial variability of population distribution, to achieve high-precision population exposure level assessment. At the same time, research has a greater development direction in the future. First, the accuracy of spatial interpolation is higher in this research. Future research can combine hybrid models such as satellite AOD data and draw distribution maps on an hourly scale to further improve the temporal and spatial resolution. Second, the majority of Weibo users are young adults and students, ignoring the situation of middle-aged and elderly people and children. The distribution map of LBS data actually shows the distribution of active users rather than the real population density. Research has shown that potential sampling bias will not weaken the performance of social media data in characterizing dynamic population distribution, so we are cautious when drawing conclusions using big data.

## 5. Conclusions

By integrating PM_2.5_ data from the National Air Quality Real-Time Monitoring Platform and introducing an improved PM_2.5_ exposure assessment model based on LBS data, this study compared and analyzed the exposure risks under static and dynamic population data and explored the potential association between lung cancer incidence and PM_2.5_ pollution levels. The study found that Guangzhou PM_2.5_ spatially presents the characteristics of high levels in the Liwan and Yuexiu urban core areas and low levels in the Conghua District and Nansha District. In the four seasons, the PM_2.5_ concentration in winter is significantly higher than that in other seasons, followed by autumn, and it is lowest in spring and summer. Based on the spatial distribution of the PM_2.5_ concentrations and PM_2.5_ population exposure intensity indicators, the population exposure risk assessment on a fine scale has been better achieved. The accuracy of dynamic exposure was determined by visual comparison, and the importance of population dynamic change in environmental exposure and health assessment was illustrated. In addition, the incidence of lung cancer is spatially similar to that of PM_2.5_ pollution, indicating a potential spatial correlation between the two. Since the smoking rate is greatly reduced at present, fine particulate matter has become the primary cause of lung cancer, and the lag effect of PM_2.5_ exposure is approximately eight years. Therefore, there is a need for more control over highly populated and highly polluted areas, for example, it is possible to set up locations in long-term high-density crowds to conduct pollution monitoring and survey work, control local vehicles according to crowd exposure levels, and achieve the most efficient resource allocation to more effectively reduce overall pollution exposure and protect public health. The results and methods discussed in this article are suitable for high-resolution population exposure studies, which can provide ideas for environmental exposure and health assessment in other typical regions. They can also provide a theoretical basis for current hotspot exposure studies. This plays a key role in formulating regional air pollution policies, realizing population health monitoring and travel health prevention and control.

## Figures and Tables

**Table 1:** Summary statistics for the mobile monitoring sampling data.

Date	Tianhe	Yuexiu	Zengcheng	Huangpu	Conghua	Panyu
23 September	17.74 ± 4.54	14.92 ± 9.18	17.334 ± 3.81	14.793 ± 5.88	12.386 ± 4.29	19.97 ± 7.88
24 September	20.74 ± 9.33	17.98 ± 8.17	17.641 ± 5.73	23.339 ± 7.34	16.519 ± 6.17	40.50 ± 10.36
25 September	19.21 ± 3.88	24.85 ± 8.95	21.285 ± 7.55	25.841 ± 5.71	26.913 ± 7.22	30.22 ± 4.77
26 September	45.72 ± 9.72	39.80 ± 7.93	35.033 ± 9.37	40.394 ± 8.62	34.204 ± 7.06	29.25 ± 7.86
27 September	45.23 ± 5.67	42.22 ± 7.29	82.107 ± 5.06	48.62 ± 6.37	41.298 ± 5.59	49.31 ± 5.70
28 September	86.30 ± 13.56	83.33 ± 11.67	78.059 ± 11.35	72.742 ± 10.47	69.468 ± 9.34	77.23 ± 17.79
29 September	58.01 ± 3.73	64.82 ± 17.48				

## Data Availability

All relevant data sources and links have been described in Section 2.2, Section 2.5 and Supplementary Materials and are also available from the authors.

## Supplementary Materials

The following are available online at https://www.mdpi.com/article/10.3390/ijerph19052629/s1, Supplementary Material: LUR model construction and LUR modeling results; Figure S1. Scatter plot of observed and predicted PM_2.5_ concentrations derived by (a) Ordinary Kriging interpolation (b) LUR model.
