# Supplementary material for: Using Multisource Data to Assess PM2.5 Exposure and Spatial Analysis of Lung Cancer in Guangzhou, China
Source: Int J Environ Res Public Health. 2022 Feb 24;19(5):2629. doi: 10.3390/ijerph19052629 (PMC8910196; doi:10.3390/ijerph19052629)
Supplement: Supplementary file 1 [file ijerph-19-02629-s001.zip › ijerph-1594623-supplementary.pdf]

International Journal of
Environmental Research and Public Health

MDPI

Article

Using Multisource Data to Assess $PM_{2.5}$ Exposure and Spatial Analysis of Lung Cancer in Guangzhou, China

Wenfeng Fan, Linyu Xu * and Hanzhong Zheng

Supplementary Materials

Citation: Fan, W.; Xu, L.; Zheng, H. Using Multisource Data to Assess $PM_{2.5}$ Exposure and Spatial Analysis of Lung Cancer in Guangzhou, China. *Int. J. Environ. Res. Public Health* **2022**, *19*, 2629. https://doi.org/10.3390/ijerph19052629

Academic Editor: Stefano Zauli-Sajani

Received: 27 January 2022
Accepted: 21 February 2022
Published: 24 February 2022

Publisher's Note: MDPI stays neutral with regard to jurisdictional claims in published maps and institutional affiliations.

Copyright: © 2022 by the authors. Licensee MDPI, Basel, Switzerland. This article is an open access article distributed under the terms and conditions of the Creative Commons Attribution (CC BY) license (https://creativecommons.org/licenses/by/4.0/).

1. LUR Model Construction

The predictive variables required by ArcGIS to develop the LUR model were collected. The land use/land cover in 2015 (30 m × 30 m) was obtained from the Resource and Environmental Science Data Center of the Chinese Academy of Sciences (http://www.resdc.cn/, accessed on 31 December, 2021). Vectorization was used to extract the area of each land-use type in different buffer zones of the monitoring station. The road network data were obtained from OpenStreetMap Data Extracts. The population density data (1 km × 1 km) of the monitoring site were obtained from the national kilometer grid population distribution dataset in 2010. The annual average temperature and wind speed of each station were interpolated from 15 meteorological stations in Guangdong Province. The DEM part of the study area was extracted from the digital elevation data of ASTER GDEM (resolution: 30 m × 30 m) provided by the Geospatial Data Cloud platform (http://www.gscloud.cn/, accessed 31 December, 2021). These geospatial variables were extracted from the 300 m to 5000 m circular buffer area around each air monitoring station to indicate the distribution of the land use and road network in the neighborhood.

Taking the annual average concentration of $PM_{2.5}$ at 28 major air quality monitoring stations in Guangzhou in 2015 as the dependent variable, multiple regression analysis was performed by integrating 49 variables of the following six indicators: the land-use type/area, road network length, population density, annual average temperature, and the wind speed and elevation at the location of the station. The independent variables of the road conditions and land use were described by the length of the road and the area of the different land types in buffers of different radii. The radius of each station was set as five types of buffers: 300 m, 500 m, 1000 m, 2000 m, and 5000 m. The land-use types covered six categories: cultivated land, woodland, grassland, water area, construction land, and unused land. To simplify the analysis, unused land was merged into construction land. The road network types were classified into four categories: highways, arterial roads, first-class roads, and other roads. Subsequently, bivariate correlation analysis was performed on all the independent variables. The independent variables that were significantly correlated with the site $PM_{2.5}$ concentration ($p < 0.05$) were selected from this process and used for stepwise regression in SPSS 13.0 with the site $PM_{2.5}$ concentration. The explanatory variables retained in the model both have a significant correlation while there is no serious multiple collinearity ($R \geq 0.7$). In ArcGIS, the city was divided into 5 km × 5 km grid points, and the regression model was used to predict the concentration of each grid point; this was followed by ordinary kriging spatial interpolation (0.5 km × 0.5 km). A simulation map of the spatial distribution of the $PM_{2.5}$ concentration in Guangzhou was subsequently derived.

2. LUR Modeling Results

After the correlation analysis of all the independent variables, the four buffer types of highway independent variables that did not meet the prior assumptions, and the

variables in varying types with multicollinearity, were eliminated. Finally, 22 out of 49 predictors were screened out for stepwise regression with the site $PM_{2.5}$ concentration.

$C_{PM2.5}$ = 0.396 × [first-class_highway_1000 m] + 0.669 × [construction _land_2000 m] + 30.029; adjusted R^2 = 0.750.

Similarly, using the ordinary Kriging method to interpolate the $PM_{2.5}$ data collected from 28 major stations in 11 administrative regions of Guangzhou, the accuracy of the two methods were compared through cross-validation (Figure S1), with the R^2 of the Kriging method and LUR model being 0.642 and 0.610, respectively. The RE and RMSE of the LUR model and the Kriging method were 0.061 and 0.057 and 3.269 and 2.967, respectively, which suggested that the errors of both were within the acceptable range. The predictability of the two models is similar. The findings overall prove that the ordinary Kriging estimated concentrations can basically represent the spatial distribution pattern of the $PM_{2.5}$. The LUR does not have a high accuracy level on the urban scale and in a short-term duration of exposure. Therefore, in the following parts the Kriging interpolation method was used to predict the $PM_{2.5}$ pollution level.

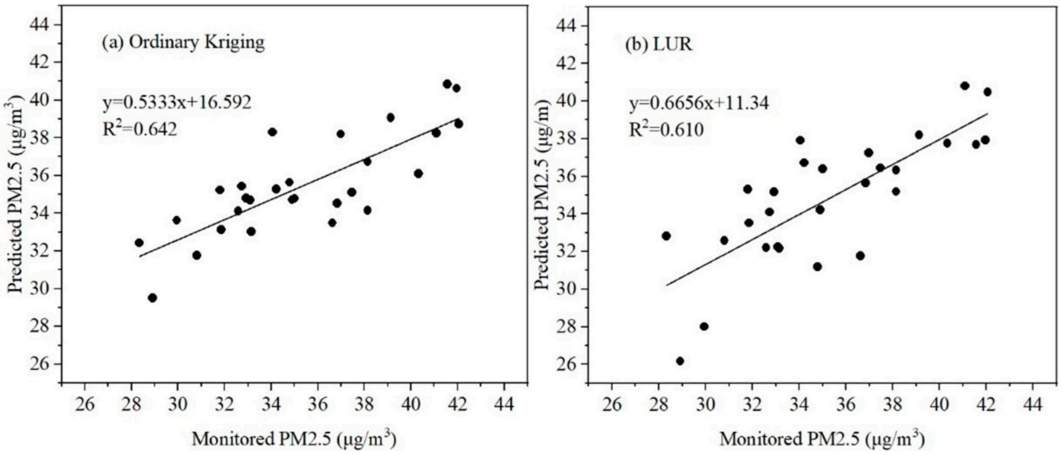

Figure S1. Scatter plot of observed and predicted $PM_{2.5}$ concentrations derived by (**a**) Ordinary Kriging interpolation (**b**) LUR model.
